# Reference intervals of thyroid hormones in Khartoum, Sudan

**DOI:** 10.1186/s13104-018-3840-5

**Published:** 2018-10-12

**Authors:** Imad R. Musa, Nagi I. Ali, Sittana A. Elseed, Osman E. Osman, Ishag Adam

**Affiliations:** 1 King Abdu Aziz Armed Forces Hospital at Air Base, Dhahran, Kingdom of Saudi Arabia; 2 Sudan Atomic Energy Commission, P. O. Box 3001, Khartoum, Sudan; 3 Department of Radiological Sciences and Medical Imaging, College of Applied Medical Sciences, Majmaah University, Majmaah, 11952 Saudi Arabia; 4 Faculty of Medicine, Alneelain University, Khartoum, Sudan; 5 Faculty of Medicine, University of Khartoum, P. O. Box 102, Khartoum, Sudan

**Keywords:** Thyroid, TSH, T3, T4, Sudan, Radioimmunoassay

## Abstract

**Objectives:**

This study aimed to establish the reference intervals (RIs) of thyroid function test among the adult Sudanese population in Khartoum, Sudan. A multi-stage survey stratified sampling method was used. Total triiodothyronine (TT3), total thyroxine (TT4) level and thyroid stimulating hormone (TSH) levels were measured using radioimmunoassay gamma counter (Riostad, Germany) to determine the reference intervals.

**Result:**

A total of 390 adults aged 20–75 years (male: 40.5%, female: 59.5%) were recruited. The median (95% intervals) serum TSH, TT4 and TT3 levels were 1.2 (0.50–3.1) mIU/L, 103.0 (63.0–159.0) nmol/L and 1.4 (0.8–2.7) nmol/L respectively. Compared with males; females had significantly lower TSH level and significantly higher TT4 level, but there was no significant difference when the TT3 level was assessed. While there was no significant difference in the level of TSH and T3 in the age group, T4 levels have shown a progressive increase with age. In summary the RIs for TSH, TT4 and TT3 in this setting were different from the levels provided by the manufacturers. A significant different was observed in TSH and FT4 when considering gender issue. The RIs were not different in the different age groups except for FT4.

## Introduction

Thyroid hormones are considered routine biochemical indices to assess and to diagnose thyroid functional disorders. It includes thyroid stimulating hormone (TSH), total triiodothyronine (T3), free triiodothyronine (FT3), total thyroxine (T4) and free thyroxine (FT4) [1]. Among these hormones, TSH is the most sensitive marker for thyroid dysfunction with a very important index for diagnosing subclinical thyroid functional diseases [2]. Besides, it has an essential role in adjusting the dose for treating both hyperthyroidism and hypothyroidism. Likewise it has a prognostic importance for tumor recurrence [3]. Hence it is wise to maintain lower levels of TSH in order to reduce its stimulatory effects on thyroid tissue. The National Academy of Clinical Biochemistry (NACB) has recently pointed to the influence of many factors that might affect thyroid function tests: physiological factors (age and pregnancy), pathological factors (hospitalization and comorbidities), medications and iodine nutritional status on thyroid test values [4, 5]. It is recommended to consider these factors when issuing these guidelines in clinical practice [4]. The laboratory methods used for processing thyroid function tests may affect the final result  [6]. In fact, most clinical laboratories are still using the reference intervals (RIs) as recommended by the commercial assay manufacturers, which is promoted as a major barrier for the accurate and high-quality diagnosis of thyroid functional diseases [7]. Iodine status has its own influence on RIs for TFT. The World Health Organization (WHO), reported that more than 2.2 billion people from 130 countries are at risk for iodine deficiency disorders (IDD) [8]. Sudan is not exempted from IDD despite the fact that IDD control programs were initiated as early as the mid1970s [9, 10].

There is a need for establishing national reference intervals for thyroid function tests. Different TSH cut-off limits have been reported in population-based studies conducted in various countries [11]. The common practice in Sudan is still adopting the reference intervals (RIs) of thyroid function tests as suggested by the commercial assay manufacturers. The current study was conducted to establish the RIs of thyroid hormones in Khartoum, the capital of Sudan and to set reference intervals for thyroid function tests which will improve accuracy of diagnosing thyroid functional disorders.

## Main text

### Methods

A multi-stage survey was conducted in Khartoum, the capital of Sudan. A total of 390 adults Sudanese participants aged 20–75 years (male: 40.5%, female: 59.5%) were recruited as reference population for this study with an estimated response rate above 80%. Subjects with a history of thyroid disease, pregnant or breastfeeding women, those with moderate-to-severe ill health and subjects who were receiving medication that may affect the thyroid function, such as estrogen, amiodarone, anti-epileptic drugs, aspirin, glucocorticoids and excess iodine ingestion were excluded.

After signing an informed consent, all participants filled out questionnaires, that requesting information on demographic characteristics, lifestyle risk factors, family history of diseases, personal medical history and proper clinical examination to exclude goiter or palpable thyroid nodule. Based on the National Academy of Clinical Biochemistry (NACB) criteria for biochemical tests, a venous blood samples (1992) were collected and allowed to clot in plain tubes (Ningbo Greetmed Medical Instruments Co., Ltd, Ningbo, 315040 China), and the serum stored at (− 20 °C) until analyzed for measurement of TSHT T3 and T4 using Radioimmunoassay gamma counter (Riostad, Germany) and kits provided by Beijing Isotope Nuclear Electronic Co., Beijing, China. The corresponding normal levels of serum TSH, TT4 and TT3, provided by the manufacturers were 0.7–5 mIU/L, 60–160 mmol/L, 0.8–3.0 mmol/L, respectively.

#### Statistics

Data were entered in computer using IBM SPSS version 20 for Windows for analyses. Tukey’s methods were used for detecting the outliers and were removed [12]. Normality was checked using the Kolmogorov–Smirnov test and TSH, T4 and T3 levels were not normally distributed. As recommended before by The International Federation of Clinical Chemistry (IFCC) non-parametric approach to RIs was sued where the median and 95.0% intervals were used to express the values for TSH, T4 and T3 [13]. The level of TSH, T4 and T3 were compared between male and females and between the different age groups using (non-parametric tests) Mann–Whitney U and by Kruskal–Wallis H, respectively. P < 0.05 was considered significant.

### Results

After removing the outliers (56) the data of 390 subjects were analyzed. Of the 390, 232 (59.5%) were females. As mentioned above the level of all the three investigated hormones was not normally distributed.

The median (95% intervals) of serum TSH, T4 and T3 levels were 1.2 (0.50–3.1) mIU/L, 103.0 (63.0–159.0) nmol/L and 1.4 (0.8–2.7) nmol/L respectively, Table 1.

**Table 1:** Median (95% intervals) of serum TSH, T4 and T3 levels according to age and gender in Khartoum, Sudan

Age groups, in years	Number	TSH, mIU/L	T4, nmol/L	T3 mIU/L
20–30	63	1.3 (0.5–3.5)	71.0 (29.8–81.0)	1.4 (0.8– 2.6)
31–40	61	1.3 (0.5–3.3)	87.0 (82.0–91.0)	1.4 (0.8–2.3)
41–50	100	1.4 (0.5–3.1)	98.0 (92.0–106.9)	1.4 (0.8–2.7)
51–60	91	1.2 (0.4–2.8)	121.0 (109.0–130.0)	1.5 (0.8–2.7)
> 60	75	1.0 (0.4–2.8)	148.0 (132.8–185.2)	1.5 (0.9–2.5)
P		0.124	< 0.001	0.182
Gender				
Male	158	1.7 (0.5–3.4)	96.5 (62.0–148.2)	1.5 (0.8–2.9)
Female	232	1.1 (0.5–3.0)	106.0 (63.0–165.0)	1.4 (0.8–2.4)
P		< 0.001	< 0.001	0.161
Total	390	1.2 (0.50–3.1)	103.0 (63.0–159.0)	1.4 (0.8–2.7)

**Fig. 1:**
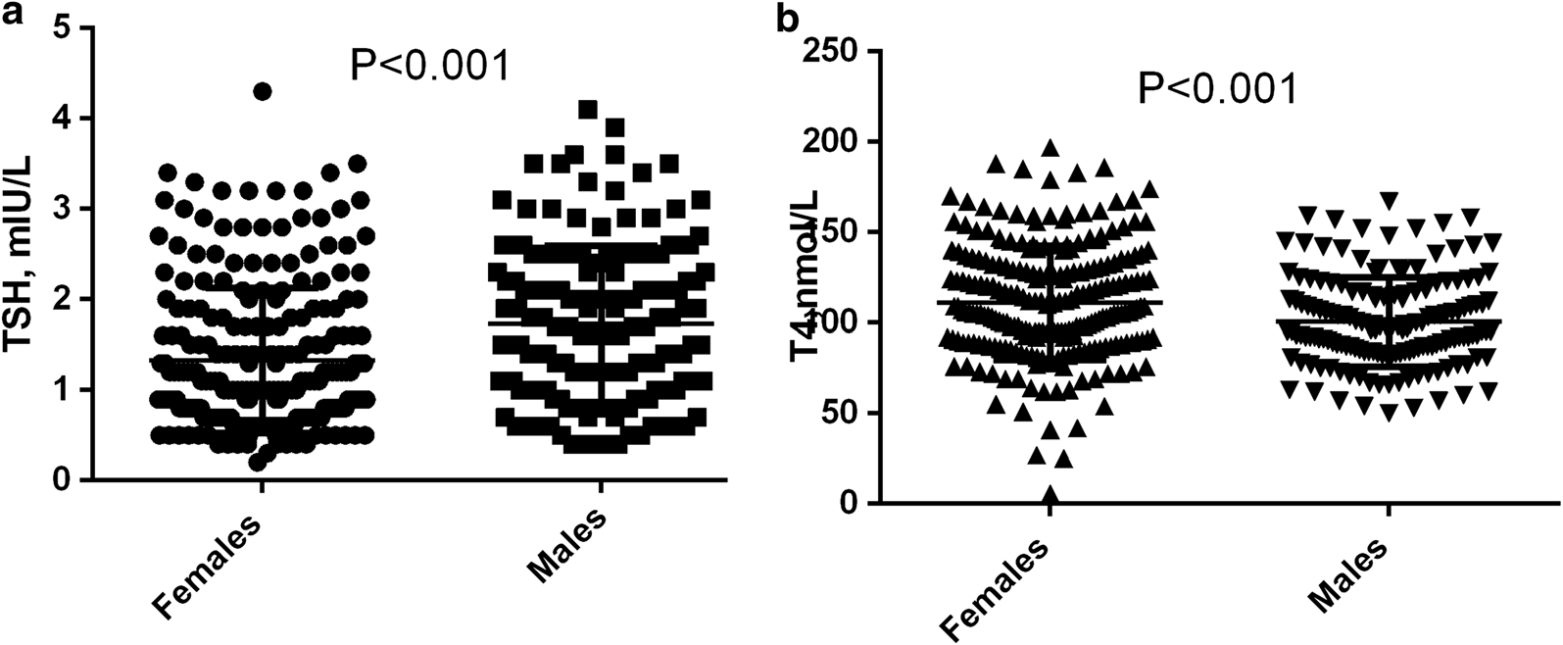
**a**, **b** Comparing TSH and T4 between males and females

While there was no significant difference in the T3 level between male and females; compared with males; females had significantly lower TSH levels and significantly higher T4 levels, Table 1, Fig. 1a and b.

**Fig. 2:**
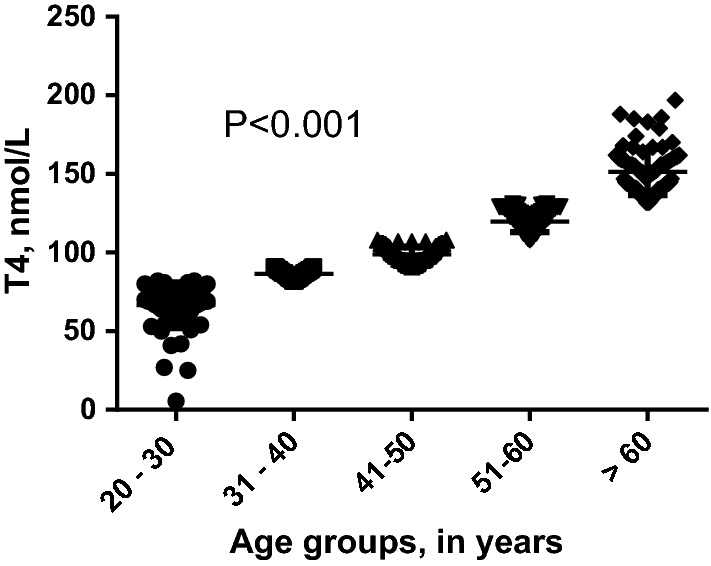
ComparingT4 between the different age groups

While there was no significant difference in the level of TSH and T3 in the age group, T4 levels have shown a progressive increase with age, Table 1, Fig. 2.

### Discussion

A different median (95% intervals) of serum TSH, T4 and T3 levels were obtained in the current study which were 1.2 (0.50–3.1) mIU/L, 103.0 (63.0–159.0) nmol/L and 1.4 (0.8–2.7) nmol/L respectively. This goes with our recent study which was conducted in western Sudan and achieved new RIs for the thyroid function tests: TSH (0.50–3.0), T4 (72.0–161.0) and T3 (0.8–2.8) [14]. Surprisingly, both studies reported slightly higher TSH levels than that obtained among pregnant women in Sudan (TSH 0.079–2.177 IU/ml) [15]. The RIs for TSH in the current study (0.50–3.1) were different and lower than that was given by the manufacturer (TSH 0.7–5 mIU/L). The same findings were obtained in different population across the world for reference intervals of TSH; In China, Northeast Germany, Japan, Mexico and Finland, RIs for TSH were (0.43–5.51 mIU/L), (0.49 –3.29  mIU/l) (0.44–4.93 mIU/L), (0.71–4.88  mIU/l) and (0.4–3.4 mU/L) respectively [7, 16–19]. In contrast to these findings, no significant difference was obtained regarding the mean serum TSH level in one clinical study [17]. Likewise, some clinical data documented a difference in reference intervals for TT3 and TT4 from that was recommended by the manufacturer although this was not obtained in the current study [7, 20, 21]. Iodine status is a major factor that has its own influence on thyroid function and may explained the variation in the reference intervals in different studies. Adding to this, the relationship between iodine intake and developing thyroid disease is U-shaped [22]. Hence both, iodine deficiency or iodine more than needed has potential effects on determining the reference intervals (RIs) of thyroid hormones among these subjects [7]. Subjects living in regions with iodine deficiency, tend to have lower reference intervals for serum TSH levels than those in regions where iodine is sufficient: large parts of Europe where most countries are inherently iodine deficient [23], reveal a considerable lower reference of internals [24, 25]. In contrast, to what is mentioned above, a higher reference of intervals for TSH, is observed in North America [26] and East Asia where iodine adequacy is observed [17, 27]. Moreover, racial variation may affect the reference intervals as supported by one study that showed that the median TSH value and reference limits were lower in African Americans and non-Hispanics than in white American [28]. Likewise, a shift towards higher TSH concentrations and reference limits with age were obtained in an Ashkenazi Jewish population, which were related to the presence of two single nucleotide polymorphisms (SNPs) in the regulatory/enhancer region of the TSH receptor gene [29].

Our study also found no significant difference in the T3 level between males and females. Interestingly, females had significantly lower TSH levels and significantly higher T4 levels than males. This was supported by Cai et al. [7] who pointed to gender variation when they reported a higher mean FT3, FT4 and T3 levels but lower mean TSH levels (1.38 mIU/L vs. 1.69 mIU/L), in males than females. Another study, showed the reference range for serum TSH levels was 0.64–8.24 mIU/L in all subjects, 0.7–8.95 mIU/L in women, and 0.56–7.15 mIU/L in men [17]. Interestingly, a recent study from Sudan, documented no significant difference in RIs for TFT, when gender issue was considered [14]. The gender as a culprit for variation in the results may be explained by the fact that TSH levels are controlled by estrogen, genetic susceptibility and environmental factors [21, 30, 31]. Hence females are more vulnerable to autoimmune thyroid disorders than males. In contrast to these findings, gender associated differences in mean values were observed for Total T4, FT3, and Total T3, but not for TSH and FT4 [20]. On the other hand, some clinical data showed no significant difference was reported for mean values of all thyroid hormones, [14] or for TSH levels [18]. Furthermore, another study showed no definitive sex-specific differences, when both serum TSH and FT3 concentrations were assessed [25].

The current study reported no significant difference in the level of TSH and T3 in the age groups, but T4 levels showed a significant progressive increase with age. We have recently reported (in Western Sudan) a higher of TSH levels among younger cases (age 31–40); while both T3 and T4 increased with progress in age [14]. Cai et al. [7] reported no significant difference in the level of TSH. Surprisingly, another study showed that, with increased age, serum FT4 levels were slightly lower in men when were compared to women [25]. Generally the effect of age on TFT is controversial as some studies demonstrated an increase in serum TSH reference limits with increasing age, [32–35] whereas in other studies serum TSH reference limits decreased with aging [25, 36]. The discrepant findings of thyroid hormones among different age groups could be explained by iodine status [25, 28, 32, 33] in the different settings [34] and genetic factor [29].

### Conclusion

The RIs for TSH, T4 and T3 in this setting were different from the levels provided by the manufacturers. The RIs were different in the different age groups and no significant gender difference was document when considering TSH and TT3.

## The limitations of the study

The total levels rather than free T3, free T4 were investigated. Thyroid antibodies and urinary iodine were not investigated. The clinical judgment and not the ultrasound was used for the evaluation of the thyroid gland.

## Abbreviations

IDD: iodine deficiency disorders
NACB: National Academy of Clinical Biochemistry
RIs: reference intervals
TFT: thyroid function test
TT3: total triiodothyronine
TT4: total thyroxine
TSH: thyroid stimulating hormone (TSH)
WHO: World Health Organization
